# Assessment of renal function indexes in methamphetamine or tramadol intoxication adults to the emergency departments: a systematic review and meta-analysis

**DOI:** 10.1186/s12873-023-00855-1

**Published:** 2023-08-11

**Authors:** Alireza Amanollahi, Yadollah Mehrabi, Mohsen Sedighi, Hamed Basir Ghafouri, Amir Zahedi, Shahin Shadnia, Koorosh Etemad

**Affiliations:** 1 Department of Epidemiology, School of Public Health and Safety, Shahid Beheshti University of Medical Sciences, Tehran, Iran; 2 Trauma and Injury Research Center, Iran University of Medical Sciences, Tehran, Iran; 3 Department of Emergency Medicine, School of Medicine, Iran University of Medical Sciences, Tehran, Iran; 4 Department of Environmental Health Engineering, Shoushtar Faculty of Medical Sciences, Shoushtar, Iran; 5 Department of Clinical Toxicology, Toxicological Research Center, Loghman Hakim Hospital, School of Medicine, Shahid Beheshti University of Medical Sciences, Tehran, Iran; 6 Department of Epidemiology, School of Public Health and Safety, Shahid Beheshti University of Medical Sciences, Tehran, Iran

**Keywords:** Tramadol, Methamphetamine, Intoxication, Kidney function indexes

## Abstract

**Background:**

Renal dysfunction is one of the adverse effects observed in methamphetamine (MET) or tramadol abusers. In this study, we aimed to review articles involving intoxication with MET or tramadol to assess the occurrence of renal dysfunction.

**Methods:**

Two researchers systematically searched PubMed, Scopus, Web of Sciences, and Google Scholar databases from 2000 to 2022. All articles that assessed renal function indexes including creatine, Blood Urea Nitrogen (BUN), and Creatine phosphokinase (CPK) in MET and tramadol intoxication at the time of admission in hospitals were included. We applied random effect model with Knapp-Hartung adjustment for meta-analysis using STATA.16 software and reported outcomes with pooled Weighted Mean (WM).

**Results:**

Pooled WM for BUN was 29.85 (95% CI, 21.25–38.46) in tramadol intoxication and 31.64(95% CI, 12.71–50.57) in MET intoxication. Pooled WM for creatinine in tramadol and MET intoxication was respectively 1.04 (95% CI, 0.84–1.25) and 1.35 (95% CI, 1.13–1.56). Also, pooled WM for CPK was 397.68(376.42-418.94) in tramadol and 909.87(549.98-1269.76) in MET intoxication. No significance was observed in publication bias and heterogeneity tests.

**Conclusion:**

Our findings showed that tramadol or MET intoxication is associated with a considerably increased risk of renal dysfunction that may result in organ failure.

## Background

Amphetamines refer to both amphetamine (AMPH) and methamphetamines (MET) that are used extra-medically. The main effect site of amphetamines is the central nervous system (CNS) and they can induce an increased sense of alertness, heightened energy and curiosity, elevated mood and attention, and increased interest in environmental stimuli [1]. Acute and long-term MET consumption may result in abnormal findings on examination of the body systems including cardiovascular, CNS, gastrointestinal, and skin [2]. Direct effects of MET and its active metabolites on renal function have been rarely reported. However, acute renal failure (ARF) attributed to MET use is often associated with hyperthermia and/or hemodynamic instability [3]. MET also may cause rhabdomyolysis and related nephropathies such as necrotizing angiitis, acute interstitial nephritis or tubular necrosis, resulting in renal injury [4, 5].

Tramadol, a synthetic opioid analgesic, is widely used across the world [6] and its primary adverse effects are nausea and vomiting, vertigo, fatigue, dry mouth, sweating, and orthostatic hypotension [7]. Tramadol stimulates presynaptic release of serotonin and suppresses serotonin reuptake, resulting in serotonin syndrome [8]. Tramadol overdose can lead to seizure, increase in Creatine phosphokinase (CPK), acute RF, hepatic failure, cardiac arrhythmias and dysfunction [9, 10]. Renal complications attributed to tramadol overdose have been rarely reported [11] and it seems that tramadol-induced seizure can cause renal failure, resulting in elevation of Blood Urea Nitrogen (BUN) and creatinine levels [12].

Regarding the increased use of MET and tramadol in recent years and lack of information about pooled effect size in renal dysfunction, we aimed to conduct a systematic review and meta-analysis of published studies related to renal dysfunction induced by tramadol or MET intoxication in patients presented to the emergency department (ED).

## Methods

### Information sources and search

We conducted comprehensive research to retrieve all published literature on the topic. For this purpose, we selected the databases\search engine of Scopus, Web of Science, PubMed, and Google Scholar. Also, to deepen the search for related literature, besides reference lists of the included studies, the citations of each selected article in Google Scholar were examined to find possible related articles. The search was performed up to 15 May 2022. We applied two restrictions on the search and selection of studies. The first restriction was in the selection of English-language articles, and the second restriction was the date of publication, which included meta-analysis studies published after 2000. In writing the strategy search, we used both free and controlled (Medical Subject Headings (MeSH) terms. The sample terms of search strategies were as follows: (amphetamine, methamphetamine, tramadol, toxic, overdose, abuser, intoxication, creatine, creatine phosphokinase, creatine kinase, urea, blood urea nitrogen).

### Eligibility criteria

The research question in the PICO structure for this review includes; (Patients: intoxicated patients referred to the hospital emergency department), Intervention/Exposure: use of tramadol or MET, Comparison; there is no comparison group in this review, Outcomes; the values of renal function indexes including BUN, creatine, and CPK. Creatinine and BUN are nitrogenous end products of metabolism that are handled primarily by glomerular filtration. They are screening tests of renal function and are valuable in evaluating renal disease. CPK is an intracellular element released by the muscular cell wall into the bloodstream following damage. Many conditions can cause derangement in CPK levels, including rhabdomyolysis, heart disease, kidney disease, or even certain medication. Elevated CPK levels are repeatedly associated with acute renal failure and the need for renal replacement therapy in patients with rhabdomyolysis. We excluded studies with individuals or addicts who have not been intoxicated or existed for other research purposes, case study design, animal studies and children’s study population, and narrative and systematic articles.

### Study selection, data items, and Data Collection

Studies were selected by observational design (prospective, retrospective), cross-sectional and case series. In the screening step, duplicate documents were initially managed, and then the recovered documents were screened in terms of the title and abstracts of the published paper. All the literature review, article selection, and data extraction were done by two independent reviewers (A. A, and A. Z). Kappa agreement rate was 84% between reviewers. Disagreements about study selection were resolved via consensus or consultation with third reviewer (K.E). All procedures were performed without blinding and the reviewers were aware of the study information. Relevant information was extracted using a customized datasheet. Basic information (first author, publication year, country, study design, intoxication agent, mean age, sex, and sample sizes) and clinical outcomes of poisoned patients including BUN, creatinine, and CPK at the admission time to ED were extracted. The reviewers contacted authors of eligible studies when additional data was needed.

### Risk of bias of included studies

The methodological qualities of included studies were evaluated by two independent reviewers (H.BG and M.S). The Newcastle Ottawa Scale (NOS) was used to evaluate the quality of the methodology [13]. This tool consists of 7 questions in 3 sections (selection, comparability, and outcome) including representativeness of the sample, sample size, non-respondents, ascertainment of the exposure, comparability, assessment of the outcome, statistical test. The quality of each NOS item is marked with a star and total awarded stars (up to ten) indicated the quality of methodology in selected studies. Assessing risk of bias for included studies was based on the JBI critical appraisal tools [14].

### Synthesis of results

The mean pooled estimation of renal function indexes was assessed using weighted mean (WM) with a 95% confidence interval (CI). We used random effect model with Knapp-Hartung adjustment method in all analyses for meta-analysis [15]. Knapp-Hartung is used to estimate uncertainty in meta-analysis when the number of studies is small and heterogeneity between studies is low. If the unit of clinical indexes was different, it was converted to a common unit (mg/dl). Also, if the outcomes in the studies were reported in different forms (median and interquartile range (IQR) or mean and standard error (SE), they were converted to mean and standard deviation (SD). Heterogeneity was estimated according to the I^2^ and chi-square tests and the existence of publication bias was assessed by Egger’s and Begg’s test. Meta-regression and sensitivity analysis were performed to find the source of heterogeneity and robustness of results. Also, GRADE (Grading of Recommendations Assessment, Development and Evaluation) was reported for assessing certainty or confidence in the effect size [16]. The STATA version 16 was used for statistical analysis.

## Results

### Study selection and characteristics

**Fig. 1:**
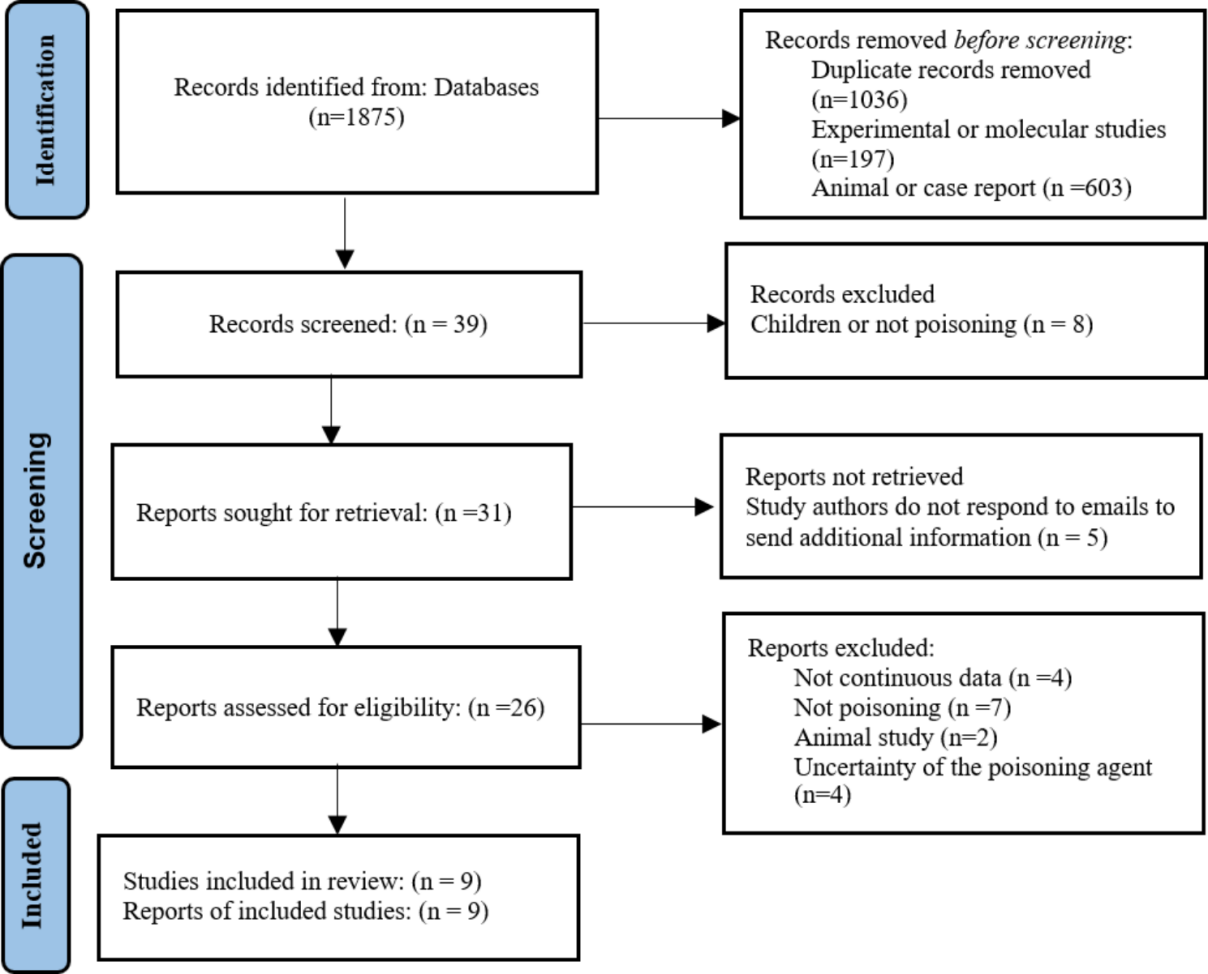
PRISMA flow diagram of the study selection

After removing duplicated studies from the various research databases, 1875 documents remained. For the screening based on study hypotheses, it was necessary to read the full text for the final decision in 31 studies, and 8 studies remained finally after this process. Moreover, one related study was included by reviewing citations received from Google Scholar, which included a total of 9 studies to extract data. Figure 1 shows the screening and selection processes of studies.

Figure 1. PRISMA flow diagram of the study selection.

Demographic information of the studies is described in Table 1. According to the table, there were 4 studies for intoxication with tramadol and 5 studies for MET. The sample size of the total study population was 1884, with an average age of 29.95. Among the papers, 5 studies were conducted in Iran [8, 12, 17–19], and 4 studies were done in other nations including United States [20], Australia [21], Thailand [22], and Egypt [23]. In all studies, the percentage of intoxicated men was higher than women. The characteristics of the included studies in the meta-analysis are summarized in Table 1. The results of the methodological quality assessment estimated by NOS were between 7 and 10 stars, which were from acceptable to good quality.

**Table 1:** Demographic information of studies included in the meta-analysis

Study	Year	Country	Study type	Intoxication agent	Age	Sex	Sample size	Initial dose	Participants	Score of quality assessment*		
										Selection	Comparability	Outcome
BahramiMotlagh (17)	2018	Iran	Cross-sectional	MET	31.70 ± 9.1	66(94)M 5(6)F	70	3.7 ± 3.1 g	Dec 2012 and July 2014-Blood sampling was at the arrival time in hospital.	****	**	*
Ismail (23)	2018	Egypt	Cross-sectional	Tramadol	27.12 ± 8.45	89(89)M 11(11)F	100	1.66 ± 0.4 g	July 2016 to Feb 2018- Patients with history of chronic liver or renal disease were also excluded.	*****	**	**
Isoardi (21)	2020	Australia	Prospective observational series	MET	30.75 ± 1.2	41(85)M 7(15)F	48	NA	Dec 2017 to Sep 2018- Baseline blood test.	****	*	***
Mohammadpour (12)	2019	Iran	Cross-sectional	Tramadol	24.75 ± 6.02	82(68)M 39(32)F	121	433.1 ± 379.5 g/ml	Mar 2016 to Mar 2017- Initial blood sample, these people also did not have any particular renal and heart failure.	****	**	*
Rahimi (19)	2014	Iran	Retrospective descriptive	Tramadol	23.7 ± 6.9	111(77)M 33(23)F	144	1971.3 ± 233.4 mg	Jan to April 2012- Laboratory findings on admission time. co‑ingestion, unknown dose, onset seizure before admission, past medical history of epilepsy or drug/substance abuse were exclusion	*****	**	**
Rahimi (18)	2018	Iran	Cross- sectional	MET	32.9 ± 10.9	174(77)M 52(23)F	226	1.64 ± 1.59 g	April 2011 to Mar 2014- Baseline laboratory results-The patients with co-ingestion, or those discharged against medical advice were excluded.	*****	*	**
Richards (20)	2020	USA	Retrospective review	MET	43.0 ± 12	676(71)M 281(29)F	957	NA	July 1, 2012, to July 1, 2017-Initial time blood sample	*****	**	***
Suriyaprom (22)	2011	Thailand	Cross-sectional	MET	33.0 ± 1.8	60(100)M	60	NA	During 3 years, venous blood sample 12 h after admission, excluded any chronic illness or other than MA substance dependence.	****	**	**
Tashakori (8)	2010	Iran	Case series	Tramadol	22.6 ± 7.4	99(63)M 59(37)F	158	1490.3 ± 1234.7 mg	Sep 1, 2006 to Aug 31, 2007- Initial biochemistry results, excluded with a history of seizure, cardiac, or kidney disorder.	*****	**	**

*Newcastle-Ottawa quality assessment scale, NA; Not Available

The risk of bias assessment based on the JBI checklist is reported in Table 2.

**Table 2:** Assessment of the risk of bias in included studies based on JBI checklist

JBI items	BahramiMotlagh (17)	Ismail (23)	Isoardi (21)	Mohammadpour (12)	Rahimi (19)	Rahimi (18)	Richards (20)	Suriyaprm (22)	Tashakori (8)
Clear criteria for inclusion	Clear	Clear	Clear	Clear	Clear	Clear	Clear	Clear	Clear
Condition measured in a standard, reliable way for all participants	Clear	Clear	Clear	Clear	Clear	Clear	Clear	Clear	Clear
Valid methods used for identification of the condition	Clear	Clear	Clear	Clear	Clear	Clear	Clear	Clear	Clear
Consecutive inclusion of participants	Clear	Clear	Clear	Clear	Clear	Clear	Clear	Clear	Clear
Complete inclusion of participants	Clear	Clear	Clear	Clear	Clear	Clear	Clear	Clear	Clear
Clear reporting of the demographics of the participants	Clear	Clear	Clear	Clear	Clear	Clear	Clear	Clear	Clear
Clear reporting of clinical information	Clear	Clear	Clear	Clear	Clear	Clear	Clear	Clear	Clear
Outcomes or follow up results of cases	Clear	Clear	Clear	Clear	Unclear	Clear	Clear	Clear	Clear
Clear reporting of the presenting site(s)/clinic(s) demographic information	Clear	Clear	Clear	Clear	Clear	Clear	Clear	Clear	Clear
Statistical analysis appropriate	Clear	Clear	Clear	Unclear	Clear	Clear	Clear	Clear	Clear
Overall appraisal	Include	Include	Include	Include	Include	Include	Include	Include	Include

### Synthesis of results and risk of Bias

#### Overall estimation of renal dysfunction

The pooled estimate of WM for renal function indexes is shown in Table 3, indicating that estimated WM for BUN (31.64 vs. 29.85), creatinine (1.35 vs. 1.04), and CPK (909.87 vs. 397.68) was higher in patients with MET intoxication compare to tramadol.

#### Heterogeneity and publication bias

Heterogeneity was not seen in all meta-analyses (I^2^ = 0.0%, and chi-square p > 0.05) except in estimated BUN for MET intoxication. Publication bias was calculated with Egger’s and Begg’s tests and publication bias was not found (p > 0.05) (Table 3).

**Table 3:** Pooled weighted mean, heterogeneity and publication bias according to renal dysfunction test using Knapp-Hartung adjustment for random effect model

Renal clinical index	Intoxication agent	Number of studies	Weighted Mean, (95%, CI)	Heterogeneity test			Publication bias	
				I^2^%	Chi-square		Egger’s	Begg’s
BUN	Tramadol	4	29.85 (21.25–38.46)	0.0	0.705		0.949	0.99
	MET	4	31.64 (12.71–50.57)	90.03	0.001		0.073	0.308
Creatinine	Tramadol	4	1.04 (0.84–1.25)	0.0	0.821		0.546	0.734
	MET	4	1.35 (1.13–1.56)	0.0	0.530		0.185	0.99
CPK	Tramadol	2	397.68 (376.42-418.94)	0.0	0.981		0.991	-
	MET	4	909.87 (549.98-1269.76)	0.0	0.910		0.585	0.174

#### Meta-regression and sensitivity analysis

According to the results of the heterogeneity test in Table 3, the heterogeneity assumption was approved for all variables except for BUN in MET intoxication. Among the reported variables that contributed probably to heterogeneity, age was considered as a covariate but did not decrease the heterogeneity values in the meta-regression model.

**Fig. 2:**
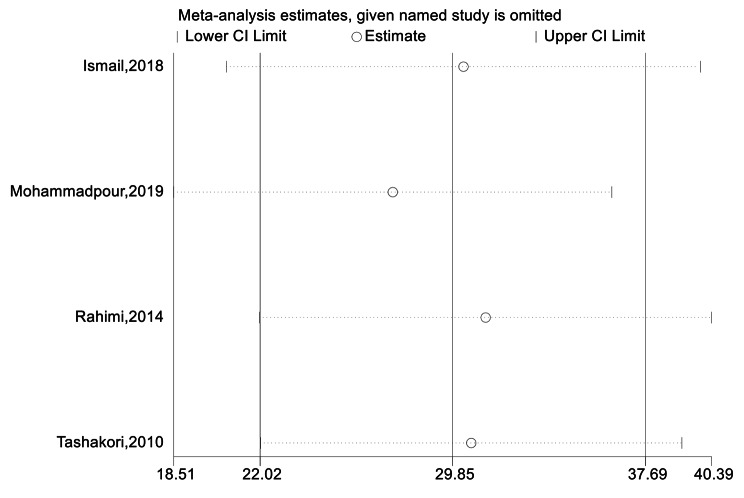
Sensitivity analysis in robustness evaluation of BUN effect size

Sensitivity analysis showed that the pooled weighted effect size in all outcomes did not depend on the effect of each study, and the overall effect size did not change after the omission of each study (Fig. 2).

#### Certainty meta-analysis

According to the GRADE approach, certainty in the evidence of observational studies in systematic review and meta-analysis started at a low level. Consequently, certainty in outcomes related to the effect size of the evidence started at a low level, as indicated in Table 4.

**Table 4:** Meta-evidence judgment based on the GRADE*

Tramadol or methamphetamine compared to no comparison group for renal dysfunction					
**Patient or population**: renal dysfunction, **Setting**: Emergency Department, **Exposure**: tramadol or methamphetamine **Comparison**: no comparison group					
**Outcomes**	**Number of participants** **(studies)** **Follow-up**	**Certainty of the evidence** **(GRADE** ^*^ **)**	**Relative effect** **(95% CI)**	**Anticipated absolute effects**	
				**Risk with no comparison group**	**Risk difference with tramadol or methamphetamine**
BUN (tramadol) assessed with: mg/dl	523 (4 observational studies)	⨁⨁⨁◯ Moderate	-	BUN ranged from **6–24** mg/dl	mean **29.85 mg/dl higher** (21.25 higher to 38.45 higher)
BUN (methamphetamine ) assessed with: mg/dl	1135 (4 observational studies)	⨁⨁⨁◯ Moderate^a^	-	BUN ranged from **6–24** mg/dl	mean **31.64 mg/dl higher** (12.71 higher to 50.57 higher)
Creatinine (tramadol) assessed with: mg/dl	523 (4 observational studies)	⨁⨁◯◯ Low	-	creatinine ranged from **0.7–1.3** md/dl	mean **1.04 md/dl higher** (0.84 higher to 1.25 higher)
Creatinine (methamphetamine) assessed with: mg/dl	1135 (4 observational studies)	⨁⨁◯◯ Low	-	creatinine ranged from **0.7–1.3** mg/dl	mean **1.35 mg/dl higher** (1.13 higher to 1.56 higher)
CPK (tramadol) assessed with: u/l	302 (2 observational studies)	⨁⨁⨁◯ Moderate	-	CPK ranged from **20–200** u/l	mean **397.68 u/l higher** (376.42 higher to 418.94 higher)
CPK (methamphetamine) assessed with: u/l	1301 (4 observational studies)	⨁⨁⨁◯ Moderate^b^	-	CPK ranged from **20–200** u/l	mean **909.87 u/l higher** (549.98 higher to 1269.76 higher)
*The risk in the intervention group (and its 95% confidence interval) is based on the assumed risk in the comparison group and the **relative effect** of the intervention (and its 95% CI). **CI**: confidence interval					
**GRADE Working Group grades of evidence** **High certainty**: we are very confident that the true effect lies close to that of the estimate of the effect. **Moderate certainty**: we are moderately confident in the effect estimate: the true effect is likely to be close to the estimate of the effect, but there is a possibility that it is substantially different. **Low certainty**: our confidence in the effect estimate is limited: the true effect may be substantially different from the estimate of the effect. **Very low certainty**: we have very little confidence in the effect estimate: the true effect is likely to be substantially different from the estimate of effect.					

Explanations

a. The amount of heterogeneity was considerable between studies.

b. The confidence interval is wide.

*GRADE, Grading of Recommendation Assessment, Development and Evaluation.

## Discussion

We performed this systematic review and meta-analysis to assess renal function indexes in tramadol and MET-intoxicated patients presenting to the ED of hospitals. The main purpose of the study was to summarize BUN, creatine, and CPK in the blood sample of intoxicated subjects. Our findings of pooled estimate analysis indicated a higher WM for BUN in tramadol and MET intoxication compared to the normal range but WM for creatinine remained within the normal range. Regarding CPK, estimated WM was notably higher in both intoxication with tramadol and MET when compared to the normal range.

Renal dysfunction is a relatively usual condition in intensive care units and several large epidemiologic investigations have reported that nephrotoxic drugs are contributing factors in 19–25% of cases of renal dysfunction in critically hospitalized patients [24]. Serum creatinine has routinely been used to diagnose renal dysfunction, which is defined as an increase in serum creatinine of at least 0.3 mg/dL or 1.5 times baseline [25, 26]. In addition, BUN is another serum marker of renal function, however, it may be altered by non-renal parameters such as catabolic condition, upper gastrointestinal bleeding, hypovolemia, and treatment with high-dose steroids [27, 28]. Serum CPK level also is another efficient biomarker for renal dysfunction in case of chemical intoxication owing to its easy availability and serial monitoring of its level throughout treatment that can predict the prognosis of patients [29].

Amphetamines-induced rhabdomyolysis has been mentioned in several case reports as an adverse effect of MET on the kidney [30]. Rhabdomyolysis is typified by the breakdown of the muscles and release of the intracellular components such as CPK, lactate dehydrogenase, and transaminases into the bloodstream [31]. The pooled WM for CPK in our meta-analysis was 909.87 for MET intoxication which was higher than the normal range (10–120 mcg/L) but lower than the reference value for rhabdomyolysis occurrence (1500 to over 100,000 units/L), indicating some degree of renal injury. Furthermore, pooled WM for creatinine and BUN in MET intoxication was 1.35 (reference range: 0.7–1.3 mg/dL) and 31.64 (reference range: 6–24 mg/dL), respectively. BahramiMotlagh et al., reported total mean CPK of 1471.1 ± 863 units/L in MET body suffers which was higher compared to our pooled WM for CPK. The reason is that some of MET body suffers in their study had ingested MET with heroin, opium, and methadone [17]. Also, mean total CPK reported by Rahimi et al. (1067.9 ± 2981.9 units/L) was higher than our pooled WM for CPK that may be because of positive history of addiction in most of the cases in their study [18]. Furthermore, they observed significant associations between agitation, seizure, and CPK level with mortality due to poisoning with amphetamines [14]. Prior investigations reported that intoxicated patients with CPK serum levels of 10,000 IU/L and higher are at increased risk of nephrotoxicity and renal dysfunction [32].

Tramadol is available in various forms with a 50 mg orally standard therapeutic dose, and 400 mg is the maximum recommended daily dose [33, 34]. Tramadol is spread to the liver, spleen, lungs, brain, and kidneys. The most frequently reported adverse effects of tramadol reported by intoxication are nausea, vomiting, CNS depression, seizure, dizziness, agitation, tachycardia, hypertension, reduced appetite, headache, itching, pruritus and rash, and gastric irritation, and skin eruption [35]. Intoxication with tramadol may also lead to multiple organ failure, coma, cardiopulmonary arrest, and death [36]. In our analysis, the pooled WM for CPK in tramadol intoxication was 397.68 which was relatively higher than the normal range. Regarding creatinine and BUN, the pooled WM respectively was 1.04 and 29.85. However, Mohammadpour et al. reported mean BUN of 38.23 ± 8.3 mg/dL in tramadol toxicity which is relatively higher in comparison with pooled WM for BUN in our analysis. In this regard, they observed the highest concentration of tramadol (491.90 vs. 374.42 QUOTE g/ml) and BUN level (53.33 vs. 23.37 mg/dl) in the seizure group compared to nonseizure cases [12]. A prior experimental investigation found only minimal renal histopathologic changes limited to tubular cells with chronic therapeutic doses of tramadol. In addition, BUN and creatinine levels remained unchanged in intoxication. However, Atici et al. reported a significant increase in creatinine and BUN levels in tramadol intoxication with seizure compared to non-seizure intoxication [11]. Tramadol consumption leads to muscle damage by causing seizures and therefore increased serum levels of CPK could be due to acute renal failure from tramadol poisoning or indirectly following seizure and rhabdomyolysis [12].

### Limitations

In our study, final analysis was substantially limited in the number and quality of studies available. The main limitation of the study is the lack of information regarding dose of consumed drugs by patients in the studies. Also, some patients in the reviewed articles may have different history of substance use, which possibly affects the results of study. Another limitation is that the level of renal index (e.g. CPK) in some cases has not been adjusted for the seizure and authors in some articles described only patients’ clinical conditions, which makes it difficult to generalize the results. Lastly, owing to the disparity of results in different countries, more comprehensive research is proposed to make a better conclusion regarding association of tramadol or MET intoxication with changes in renal function indexes.

## Conclusion

In conclusion, this systematic review and meta-analysis suggests that tramadol and MET intoxication is associated with a significant rise in renal function indexes. Moreover, our pooled analysis showed that WM of CPK was higher mainly in MET poisoners compared to tramadol. Through this study we indicated that excessive use and intoxication with tramadol and MET increase risk of renal dysfunction and failure. Therefore, clinicians and health care staff in EDs should suspect renal injury in poisoners with MET and tramadol, especially if routine renal indexes are abnormal.

## Data Availability

All data generated or analyzed during this study are available from the corresponding author on reasonable request.

## Declarations

### Identification code in prospero

CRD42022321523.

## Abbreviations

MET: Methamphetamine
BUN: Blood Urea Nitrogen
CPK: Creatine phosphokinase
WM: Weighted Mean
AMPH: Amphetamine
CNS: Central Nervous System
ARF: Acute Renal Failure
ED: Emergency Department
MeSH: Medical Subject Headings
NOS: Newcastle Ottawa Scale
CI: Confidence Interval
IQR: Interquartile Range
SD: Standard Deviation
SE: Standard Error
GRADE: Grading of Recommendations Assessment, Development and Evaluation
